# Supplementary material for: Deletion of wfs1 Impairs Oligodendrocyte Precursor Cells Dorsal Distribution and Myelination Through the wfs1-hmgcs1 Axis in Zebrafish
Source: Biology (Basel). 2026 Aug 16;15(16):1402. doi: 10.3390/biology15161402 (PMC13510151; doi:10.3390/biology15161402)
Supplement: Supplementary file 1 [file biology-15-01402-s001.zip › File S1-Transcriptome sequencing data and differentially expressed genes (DEGs) in wfs1-- zebrafish at 5 dpf/1.GO/wfs1KOvsWT/GSEA/WT/REGULATION_OF_CELL_SHAPE/REGULATION_OF_CELL_SHAPE(GO_0008360).html]

Details for gene set REGULATION\_OF\_CELL\_SHAPE(GO:0008360)[GSEA]


|  || Dataset | deg.sample |
| Phenotype | sample.cls |
| Upregulated in class | WT |
| GeneSet | REGULATION\_OF\_CELL\_SHAPE(GO:0008360) |
| Enrichment Score (ES) | -0.43812737 |
| Normalized Enrichment Score (NES) | -2.153519 |
| Nominal p-value | 0.0 |
| FDR q-value | 0.06103634 |
| FWER p-Value | 0.06 |
Table: GSEA Results Summary

  

Fig 1: Enrichment plot: REGULATION\_OF\_CELL\_SHAPE(GO:0008360)
  
Profile of the Running ES Score & Positions of GeneSet Members on the Rank Ordered List

  

| PROBE | DESCRIPTION   (from dataset) | GENE SYMBOL | GENE\_TITLE | RANK IN GENE LIST | RANK METRIC SCORE | RUNNING ES | CORE ENRICHMENT || 1 | 436718 | rhocb |  |  | 948 | 2.171 | 0.0171 | No |
| 2 | 553415 | rhobtb2a |  |  | 3000 | 1.035 | -0.0250 | No |
| 3 | 101883603 | cdc42ep1a |  |  | 3021 | 1.031 | -0.0033 | No |
| 4 | 553209 | brwd3 |  |  | 3183 | 0.996 | 0.0131 | No |
| 5 | 550557 | palm1a |  |  | 3646 | 0.903 | 0.0181 | No |
| 6 | 562804 | si:dkey-6b12.5 |  |  | 4441 | 0.754 | 0.0095 | No |
| 7 | 407694 | fmnl3 |  |  | 5464 | 0.636 | -0.0089 | No |
| 8 | 777623 | zgc:154093 |  |  | 5700 | 0.618 | -0.0029 | No |
| 9 | 795681 | fam171a1 |  |  | 6425 | 0.543 | -0.0140 | No |
| 10 | 80963 | rnd3a |  |  | 6599 | 0.528 | -0.0080 | No |
| 11 | 751645 | rnd2 |  |  | 6720 | 0.517 | -0.0006 | No |
| 12 | 334724 | anxa1a |  |  | 7020 | 0.490 | 0.0006 | No |
| 13 | 559824 | palmda |  |  | 7144 | 0.478 | 0.0071 | No |
| 14 | 100000814 | palmdb |  |  | 7700 | 0.440 | -0.0008 | No |
| 15 | 100006132 | si:ch211-1f22.2 |  |  | 8076 | 0.417 | -0.0036 | No |
| 16 | 100333657 | plxnb2b |  |  | 9204 | 0.353 | -0.0314 | No |
| 17 | 541404 | cdc42ep2 |  |  | 9647 | 0.324 | -0.0383 | No |
| 18 | 336933 | rhogb |  |  | 9858 | 0.312 | -0.0382 | No |
| 19 | 798858 | brwd1 |  |  | 10194 | 0.291 | -0.0424 | No |
| 20 | 573655 | rhof |  |  | 10295 | 0.285 | -0.0394 | No |
| 21 | 336613 | cyfip1 |  |  | 10479 | 0.274 | -0.0392 | No |
| 22 | 561433 | plxnb2a |  |  | 11165 | 0.235 | -0.0557 | No |
| 23 | 556424 | rhoga |  |  | 11444 | 0.220 | -0.0597 | No |
| 24 | 558920 | amotl2a |  |  | 11469 | 0.218 | -0.0557 | No |
| 25 | 561285 | plxna2 |  |  | 11803 | 0.201 | -0.0618 | No |
| 26 | 566546 | plxnb3 |  |  | 12800 | 0.150 | -0.0899 | No |
| 27 | 100332334 | palm2 |  |  | 12960 | 0.143 | -0.0918 | No |
| 28 | 794234 | cdc42se2 |  |  | 13047 | 0.137 | -0.0916 | No |
| 29 | 325938 | plxna4 |  |  | 13702 | 0.107 | -0.1098 | No |
| 30 | 550268 | zgc:110283 |  |  | 13972 | 0.094 | -0.1162 | No |
| 31 | 447829 | cdc42ep3 |  |  | 14241 | 0.081 | -0.1229 | No |
| 32 | 557062 | fmnl1a |  |  | 14435 | 0.073 | -0.1274 | No |
| 33 | 415107 | bves |  |  | 14449 | 0.072 | -0.1263 | No |
| 34 | 393573 | palm1b |  |  | 14872 | 0.052 | -0.1384 | No |
| 35 | 555454 | myh10 |  |  | 14890 | 0.051 | -0.1378 | No |
| 36 | 403050 | pard3ab |  |  | 15104 | 0.041 | -0.1436 | No |
| 37 | 567422 | plxna3 |  |  | 15131 | 0.040 | -0.1436 | No |
| 38 | 100002938 | cdc42ep4b |  |  | 15380 | 0.029 | -0.1508 | No |
| 39 | 445393 | ppp1r12a |  |  | 15499 | 0.022 | -0.1540 | No |
| 40 | 30767 | stat3 |  |  | 16064 | -0.004 | -0.1716 | No |
| 41 | 553413 | rhoh |  |  | 16095 | -0.005 | -0.1725 | No |
| 42 | 334613 | fn1b |  |  | 16618 | -0.021 | -0.1884 | No |
| 43 | 795761 | plxna1a |  |  | 17037 | -0.034 | -0.2008 | No |
| 44 | 795167 | rac3b |  |  | 17171 | -0.040 | -0.2042 | No |
| 45 | 394125 | rhoca |  |  | 17514 | -0.054 | -0.2137 | No |
| 46 | 559784 | amotl2b |  |  | 17599 | -0.058 | -0.2151 | No |
| 47 | 406411 | rhoaa |  |  | 18330 | -0.091 | -0.2361 | No |
| 48 | 324681 | rhogc |  |  | 18482 | -0.098 | -0.2388 | No |
| 49 | 561431 | myh9b |  |  | 18683 | -0.107 | -0.2427 | No |
| 50 | 792631 | si:dkey-263f15.2 |  |  | 18924 | -0.119 | -0.2477 | No |
| 51 | 327204 | rac1a |  |  | 19053 | -0.124 | -0.2490 | No |
| 52 | 492608 | fmnl2b |  |  | 19458 | -0.142 | -0.2587 | No |
| 53 | 561012 | plxnb1a |  |  | 19539 | -0.145 | -0.2580 | No |
| 54 | 325042 | tnika |  |  | 19875 | -0.160 | -0.2651 | No |
| 55 | 553051 | fermt2 |  |  | 20702 | -0.199 | -0.2868 | No |
| 56 | 393653 | rhogd |  |  | 21640 | -0.248 | -0.3109 | No |
| 57 | 406659 | rhoac |  |  | 23154 | -0.338 | -0.3511 | No |
| 58 | 100006041 | rhoab |  |  | 24773 | -0.436 | -0.3926 | No |
| 59 | 405780 | rnd1a |  |  | 25857 | -0.516 | -0.4155 | No |
| 60 | 437027 | rac3a |  |  | 26579 | -0.586 | -0.4255 | Yes |
| 61 | 570222 | plxnb1b |  |  | 26891 | -0.619 | -0.4219 | Yes |
| 62 | 565844 | rhobtb2b |  |  | 26936 | -0.623 | -0.4098 | Yes |
| 63 | 791998 | cdc42se1 |  |  | 26951 | -0.625 | -0.3967 | Yes |
| 64 | 100005469 | fn1a |  |  | 27421 | -0.659 | -0.3972 | Yes |
| 65 | 415151 | rac2 |  |  | 27490 | -0.672 | -0.3848 | Yes |
| 66 | 494158 | anxa1c |  |  | 27522 | -0.675 | -0.3712 | Yes |
| 67 | 436864 | rnd3b |  |  | 27850 | -0.722 | -0.3659 | Yes |
| 68 | 562838 | rac1b |  |  | 28146 | -0.763 | -0.3586 | Yes |
| 69 | 100007808 | cdc42ep1b |  |  | 28351 | -0.795 | -0.3479 | Yes |
| 70 | 558275 | cdc42ep5 |  |  | 28595 | -0.838 | -0.3374 | Yes |
| 71 | 100006166 | si:ch211-1f22.1 |  |  | 28986 | -0.917 | -0.3298 | Yes |
| 72 | 431730 | cdc42ep4a |  |  | 29801 | -1.164 | -0.3302 | Yes |
| 73 | 792699 | rac1l |  |  | 30004 | -1.247 | -0.3096 | Yes |
| 74 | 100006456 | fmnl2a |  |  | 30146 | -1.312 | -0.2857 | Yes |
| 75 | 353358 | anxa1b |  |  | 30605 | -1.592 | -0.2657 | Yes |
| 76 | 562273 | plxnc1 |  |  | 30870 | -1.828 | -0.2345 | Yes |
| 77 | 561237 | si:dkey-183c2.4 |  |  | 30932 | -1.894 | -0.1955 | Yes |
| 78 | 100034640 | si:ch211-127b11.1 |  |  | 31262 | -2.350 | -0.1550 | Yes |
| 79 | 553414 | rnd1b |  |  | 31592 | -3.235 | -0.0954 | Yes |
| 80 | 569727 | phip |  |  | 31833 | -4.848 | 0.0018 | Yes |
Table: GSEA details
[plain text format]

  

Fig 2: REGULATION\_OF\_CELL\_SHAPE(GO:0008360)
  
Blue-Pink O' Gram in the Space of the Analyzed GeneSet

  

Fig 3: REGULATION\_OF\_CELL\_SHAPE(GO:0008360): Random ES distribution
  
Gene set null distribution of ES for
 **REGULATION\_OF\_CELL\_SHAPE(GO:0008360)**
